# Supplementary material for: Empirical methods for controlling false positives and estimating confidence in ChIP-Seq peaks
Source: BMC Bioinformatics. 2008 Dec 5;9:523. doi: 10.1186/1471-2105-9-523 (PMC2628906; doi:10.1186/1471-2105-9-523)
Supplement: Additional File 1 — A variety of html documents from the USeq web site detailing the available applications, their best usage, output file type descriptions, command line menus, etc. [file 1471-2105-9-523-S1.zip › USeqUserGuides/JavaDoc.zip!/JavaDoc/util/bio/seq/Seq.html]

Seq


|  |  |  |
| --- | --- | --- |
| **Overview** \| **Package** \| **Class** \| **Use** \| **Tree** \| **Deprecated** \| **Index** \| **Help** | |  |
| **PREV CLASS**   NEXT CLASS | **FRAMES**    **NO FRAMES**     **All Classes** |  |
| SUMMARY: NESTED \| FIELD \| CONSTR \| METHOD | DETAIL: FIELD \| CONSTR \| METHOD |  |


---


## util.bio.seq Class Seq

```
java.lang.Object
  util.bio.seq.Seq
```

---

``` public class Seq extends java.lang.Object ```

For manipulating nucleic acid sequences.

---

| **Field Summary** | |
| --- | --- |
| `static java.lang.String[]` | `all4BaseCombinations`             All possible 4 base combinations. |
| `static java.util.regex.Pattern` | `chrLetter` |
| `static java.util.regex.Pattern` | `chrNumber` |


**Constructor Summary**

`Seq()`


| **Method Summary** | |
| --- | --- |
| `static java.lang.String` | `complementDNA(java.lang.String seq)`             Takes a DNA seq and complement it, ambiguous symbols OK. |
| `static int[]` | `countBases(java.lang.String sequence)`             Given a sequence returns the number of G's,A's,T's,C's,N's as an int[5]. |
| `static java.lang.String` | `extractChromosomeName(java.lang.String x)`             Attempts to extract chr1,2,3...22 or chrX,Y,M,MT from the String. |
| `static boolean[]` | `fetchGCContent(char[] chromosomeSequence)`             Converts a DNA sequence into a boolean[], everything not g or c is recorded as false. |
| `static java.lang.String` | `fetchSubSequence(int start, int stop, int bpFirstBase, java.lang.String sequence)`             Returns a sub sequence given a relative start and stop, the bp for the first base. |
| `static java.lang.String` | `fetchSubSequenceInterbaseCoordinates(int start, int stop, int bpFirstBase, java.lang.String sequence)`             Returns a sub sequence given a relative start and stop, the bp for the first base. |
| `static java.lang.String` | `filterDNASeqLeaveWS(java.lang.String seq)`             Deletes any non IUP characters but leaves whitespaces |
| `static java.lang.String` | `filterDNASequence(java.lang.String seq)`             Deletes any non IUP characters |
| `static java.lang.String` | `filterDNASequenceStrict(java.lang.String seq)`             Deletes any non GATCNX characters |
| `static java.lang.String` | `genDashes(java.lang.String seq1, java.lang.String seq2)`             Generates identitiy dashes (ie"\|\|\| \| \|\|\|") between two aligned sequences |
| `static java.util.HashMap` | `makeByte4BaseMap()`             Makes a hash map of each 4bp combination in the all4BaseCombinations String[] and a unique Byte. |
| `static java.util.HashMap` | `makeChromosomeNameFileHash(java.io.File[] files)`             Uses Seq.extractChromosomeName() to extract a chromosome name from each file. |
| `static int[][]` | `makeFrequencyMatrix(java.lang.String[] hits)`             Generates a matrix of the number of As Cc Gg Ts (top) by 1,2,3,4...positions in the motif (side) observed in all the Strings of the String[]. |
| `static java.lang.String` | `readBinarySequence(java.io.File file)`             Reads a binary sequence file returning gatc or n, lower case. |
| `static java.lang.String` | `reverseComplementDNA(java.lang.String seq)`             Takes a DNA seq and reverse comps it, ambiguous symbols OK. |
| `static boolean` | `writeBinarySequence(java.lang.String seq, java.io.File file)`             Writes a binary sequence, gatc, anything else is assumed to be n. |

**Methods inherited from class java.lang.Object**

`equals, getClass, hashCode, notify, notifyAll, toString, wait, wait, wait`

**Field Detail**

### chrNumber

```
public static final java.util.regex.Pattern chrNumber
```

---


### chrLetter

```
public static final java.util.regex.Pattern chrLetter
```

---


### all4BaseCombinations

```
public static final java.lang.String[] all4BaseCombinations
```

:   All possible 4 base combinations.


**Constructor Detail**

### Seq

```
public Seq()
```


**Method Detail**

### extractChromosomeName

```
public static java.lang.String extractChromosomeName(java.lang.String x)
```

:   Attempts to extract chr1,2,3...22 or chrX,Y,M,MT from the String.
    Returns chromosome or null.

---


### makeChromosomeNameFileHash

```
public static java.util.HashMap makeChromosomeNameFileHash(java.io.File[] files)
```

:   Uses Seq.extractChromosomeName() to extract a chromosome name from each file.
    Adds this to a HashMap containing key: value, chrom name: File.
    Returns null if two files with the same chromosome name are found.
    Skips any files in which a chromosome name cannot be extracted.

---


### fetchGCContent

```
public static boolean[] fetchGCContent(char[] chromosomeSequence)
```

:   Converts a DNA sequence into a boolean[], everything not g or c is recorded as false.
    Returns null if a fasta is not found.

---


### writeBinarySequence

```
public static boolean writeBinarySequence(java.lang.String seq,
                                          java.io.File file)
```

:   Writes a binary sequence, gatc, anything else is assumed to be n.

    :   **Returns:**: true if sucessful, false if something bad happened.

---


### readBinarySequence

```
public static java.lang.String readBinarySequence(java.io.File file)
```

:   Reads a binary sequence file returning gatc or n, lower case.

    :   **Returns:**: null if something bad happened.

---


### makeByte4BaseMap

```
public static java.util.HashMap makeByte4BaseMap()
```

:   Makes a hash map of each 4bp combination in the all4BaseCombinations String[] and a unique Byte.

---


### filterDNASequence

```
public static java.lang.String filterDNASequence(java.lang.String seq)
```

:   Deletes any non IUP characters

---


### filterDNASequenceStrict

```
public static java.lang.String filterDNASequenceStrict(java.lang.String seq)
```

:   Deletes any non GATCNX characters

---


### filterDNASeqLeaveWS

```
public static java.lang.String filterDNASeqLeaveWS(java.lang.String seq)
```

:   Deletes any non IUP characters but leaves whitespaces

---


### fetchSubSequence

```
public static java.lang.String fetchSubSequence(int start,
                                                int stop,
                                                int bpFirstBase,
                                                java.lang.String sequence)
```

:   Returns a sub sequence given a relative start and stop, the bp for the first base.
    Stop is inclusive.
    0 starts.

---


### fetchSubSequenceInterbaseCoordinates

```
public static java.lang.String fetchSubSequenceInterbaseCoordinates(int start,
                                                                    int stop,
                                                                    int bpFirstBase,
                                                                    java.lang.String sequence)
```

:   Returns a sub sequence given a relative start and stop, the bp for the first base.
    Interbase coordinates, 0 starts, stop is excluded.

---


### genDashes

```
public static java.lang.String genDashes(java.lang.String seq1,
                                         java.lang.String seq2)
```

:   Generates identitiy dashes (ie"||| | |||") between two aligned sequences

---


### countBases

```
public static int[] countBases(java.lang.String sequence)
```

:   Given a sequence returns the number of G's,A's,T's,C's,N's as an int[5].
    Any non word chars are deleted, afterward any non GATC chars are counted
    as Ns. Case, space insensitive.

---


### makeFrequencyMatrix

```
public static int[][] makeFrequencyMatrix(java.lang.String[] hits)
```

:   Generates a matrix of the number of As Cc Gg Ts (top) by 1,2,3,4...positions in the
    motif (side) observed
    in all the Strings of the String[]. The String[] should contain equal length Strings,
    comprised entirely of GATC.

---


### reverseComplementDNA

```
public static java.lang.String reverseComplementDNA(java.lang.String seq)
```

:   Takes a DNA seq and reverse comps it, ambiguous symbols OK.
    Will warn if it finds an unrecognized base. Works with ' GATCRYWSKMBDHVNX .- '
    case and space insensitive.

---


### complementDNA

```
public static java.lang.String complementDNA(java.lang.String seq)
```

:   Takes a DNA seq and complement it, ambiguous symbols OK.
    Will warn if it finds an unrecognized base. Works with ' GATCRYWSKMBDHVNX .- '
    case and space insensitive.


---


|  |  |  |
| --- | --- | --- |
| **Overview** \| **Package** \| **Class** \| **Use** \| **Tree** \| **Deprecated** \| **Index** \| **Help** | |  |
| **PREV CLASS**   NEXT CLASS | **FRAMES**    **NO FRAMES**     **All Classes** |  |
| SUMMARY: NESTED \| FIELD \| CONSTR \| METHOD | DETAIL: FIELD \| CONSTR \| METHOD |  |


---
